# Role of an Oxidant Mixture as Surface Modifier of Porous Silicon Microstructures Evaluated by Spectroscopic Ellipsometry

**DOI:** 10.1038/srep24798

**Published:** 2016-04-21

**Authors:** Zeuz Montiel-González, Salvador Escobar, Rocío Nava, J. Antonio del Río, Julia Tagüeña-Martínez

**Affiliations:** 1 Instituto de Energías Renovables, Universidad Nacional Autónoma de México, Temixco, Morelos, C.P. 62580, México; 2 CONACYT Research Fellow-Centro de Investigación en Materiales Avanzados S.C., Unidad Monterrey, Apodaca, Nuevo León, C.P. 66600, México

## Abstract

Current research on porous silicon includes the construction of complex structures with luminescent and/or photonic properties. However, their preparation with both characteristics is still challenging. Recently, our group reported a possible method to achieve that by adding an oxidant mixture to the electrolyte used to produce porous silicon. This mixture can chemically modify their microstructure by changing the thickness and surface passivation of the pore walls. In this work, we prepared a series of samples (with and without oxidant mixture) and we evaluated the structural differences through their scanning electron micrographs and their optical properties determined by spectroscopic ellipsometry. The results showed that ellipsometry is sensitive to slight variations in the porous silicon structure, caused by changes in their preparation. The fitting process, based on models constructed from the features observed in the micrographs, allowed us to see that the mayor effect of the oxidant mixture is on samples of high porosity, where the surface oxidation strongly contributes to the skeleton thinning during the electrochemical etching. This suggests the existence of a porosity threshold for the action of the oxidant mixture. These results could have a significant impact on the design of complex porous silicon structures for different optoelectronic applications.

Porous silicon (pSi) is a nanostructured material produced by the electrochemical etching of crystalline silicon (c-Si) in a hydrofluoric acid (HF) based solution. When the thickness of its pore walls (skeleton) is of a few nanometers, pSi shows visible luminescence due to the quantum confinement effect on the charge carries, mainly in samples prepared from high resistivity c-Si substrates^1^. Moreover, it is well documented that by alternating the applied electrical current during the fabrication of pSi, it is possible to produce a multilayer structure formed by layers of different porosity (refractive index). One dimensional photonic structures are usually produced from low resistivity c-Si substrates, where a high refractive index contrast between layers can be obtained^2^,^3^. Current research on pSi is concerned with the construction of complex photonic and luminescent structures for different applications, based on the especial correlation between its microstructure and optical properties, that could lead to a tuneable behaviour of light inside these materials^2^,^3^,^4^,^5^,^6^. Recently, our group reported that by adding a polyoxometalate and hydrogen peroxide as an oxidant mixture (OM) to the electrolyte, to control the thickness and surface passivation of the skeleton, it is possible to produce luminescent and photonic properties in a same pSi structure^5^. The final properties of each pSi sample depend strongly on the effect of the OM over its growth mechanism, thus a robust characterization is determinant to help in the understanding of such properties. Since the appearance of the first report about pSi, a great amount of characterization techniques, led by electron microscopy^4^, have contributed with valuable results. The observation of phenomena like luminescence, brought the optical techniques like photoluminescence, reflectometry and ellipsometry to play a very important role in determining different properties^4^,^7^,^8^. Therefore, the analysis of the relationship between the optical and structural properties of pSi, through ellipsometric measurements in the UV-Vis-NIR region of the electromagnetic spectrum, can provide important evidence to study the effect of the addition of the OM in the fabrication of pSi.

Several ellipsometric optical models have been applied to a great variety of pSi structures and after a considerable amount of debate about the reliability and validity of those models, some commonly accepted results give the guidelines that set a point of departure in the analysis of specific samples. These results were obtained mainly from two approaches that have been particularly successful: the gradual porosity^7^ and the morphological anisotropy models^9^. Each of them has advantages and disadvantages, however, the boundary of their applicability has not been well defined so far and results from other characterization techniques such as Scanning Electron Microscopy (SEM), could be determinant to complement the optical analysis based on these models.

The main goal of this work is to contribute to the understanding of the effect of the OM on the microstructure of pSi, establishing the determination of optical properties by Spectroscopic Ellipsometry (SE) as a tool to achieve this propose. We compared pSi samples produced with and without the OM in the electrolyte. The analysis of the ellipsometric data is based on two models that closely approach the particular structure shown in the SEM micrographs of each sample. The study of the optical properties determined from the SE analysis based on models designed from the SEM micrographs, help us to understand the complex relationship between the microstructure and the optical response of pSi.

The organization of this work is as follows: in section 2, we present the procedure to fabricate the pSi samples with and without the OM. Then, we argue the need of different models to represent their microstructure to determine their optical properties from SE measurements. In section 3, we discuss our results for the ellipsometric spectra and the depolarization factor. In addition, we analyze the different microstructures used to arrive to the optical properties (refractive index and extinction coefficient). Finally, we conclude with some remarks.

## Experimental Details

The pSi samples were produced by the typical electrochemical etching of p type boron doped c-Si (100) wafers with electrical resistivity of 0.01–0.02 Ω-cm^3^,^4^,^5^. As electrical contact, an aluminum film was evaporated on the back side of c-Si, then the sample was heated up to 550 °C in an inert atmosphere for 15 min. All the samples were produced under the same conditions except for the electrolyte composition. The first electrolyte was composed of HF, ethanol and glycerol in a volume ratio of 3:7:1. In the second electrolyte, one part of ethanol was replaced by the OM. The OM is composed of phosphomolybdic acid hydrate (H_3_PMo_12_O_40_) at a concentration of 10^−3 ^M in ethanol and H_2_O_2_ in a 24:1 volume ratio. The electrical currents applied to produce each pair of samples of different porosity were 6.0, 50.0 and 90.0 mA. In this way, pSi layers with approximately the same thickness (500 nm) were produce from the c-Si substrates of 1.54 cm^2^ ± 0.04 cm^2^ in area. Porous silicon samples were passivated by thermal oxidation at 100 °C during 30 min. We prepared at least three samples for each etching condition (applied electrical current and electrolyte composition). The reflectance spectra were used as reference of sample repeatability because it depends on the optical path length (refractive index and thickness). Samples corresponding to the same etching conditions show an average shift of ±10 nm in the reflectance spectrum in the visible range.

SE measurements were carried out in a phase modulated ellipsometer *Horiba Yobin Yvon DH10* at an incidence angle of 70° with a spot size of 1 mm, in a photon energy range of 1.5 to 5.0 eV with a step 0.02 eV. The software DeltaPsi 2® was used for the fitting process. Cross section micrographs of the pSi samples were obtained from a Hitachi Scanning Electron Microscope S-550 to characterize the thickness and morphology trends. Moreover, the micrographs served as a reference to design the optical models for the SE analysis.

### Ellipsometric modeling

SE measures the change of the polarization state (ρ) of light upon oblique reflection from a surface. The characteristic parameters in SE are the ellipsometric angles Ψ and Δ. These parameters are related to the complex reflection coefficients (*r*), with polarizations parallel (*p*) and perpendicular (*s*) to the plane of incidence of the system under study, through the fundamental equation of ellipsometry: ρ = r_p_/r_s_ = tanΨ exp(*i*Δ). Each reflection coefficient represents the ratio between the incident and the reflected light, which are functions of several parameters like the complex refractive indices, the angle of incidence of the polarized light and, for stratified media they also depend on the thickness of each layer^10^. SE is a nondestructive and indirect characterization technique that has been extensively applied to the study of optical and structural properties of pSi^7^,^8^. Due to their indirect nature, it is necessary to construct an optical model, describing microstructural features of the sample as accurate as possible to determine structural and optical properties. The conclusions reached so far highlight that the pSi optical properties have a very complex dependence on its microstructure, thus a careful procedure must take into account the different factors affecting its optical response. Most of these factors have been already identified and include the heterogeneous mixture of the solid phase (c-Si pore walls + surface passivation) and air, which can be represented through suitable effective medium theories^4^,^7^,^8^. In this work, the extensively used Bruggeman (EMA) and Maxwell-Garnett (MG) approaches were chosen due to the successful results reported^4^. These models work when the sizes of the heterogeneities (≈10 nm) in the pSi mixture do not exceed the limits of their applicability with respect to the quasistatic approximation (wavelengths larger than the heterogeneities of the mixture)^11^. The surface passivation of the pSi skeleton also plays an important role and although it depends on post-preparation treatments, it can be regarded as the c-Si native oxide (SiO_2_)^8^,^12^,^13^. The quantum confinement effects must be taken into account when the pore walls thickness is approximately or less than 3 nm^14^. Finally, the microscopic column-like structure, which under certain circumstances can lead to anisotropic behavior of the optical response, must be considered too^9^,^15^.

**Figure 1:**
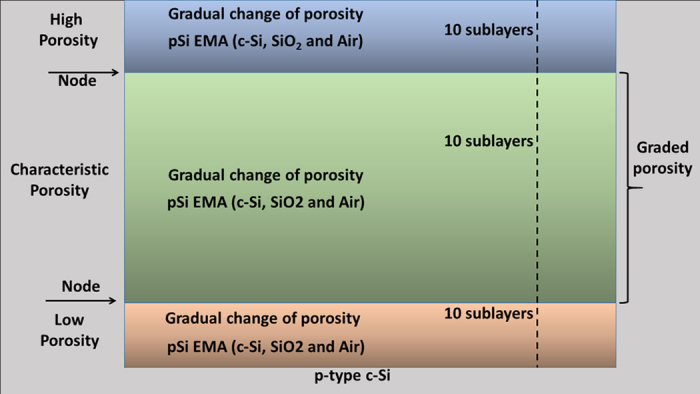
Scheme of the general structure of the gradual porosity model.

It is in the microstructural model where the conceptual difference between the two approaches used in this work resides. On one side, the gradual model takes into account the porosity gradient from top to bottom shown by pSi. This porosity gradient is modelled by three main regions: the region close to the c-Si substrate (low porosity), the middle region having the representative features of the sample (characteristic porosity) and the top region, which spends the longer time in contact with the electrolyte during the anodization (high porosity). Depending on the substrate electronic properties and the electrochemical etching conditions these regions can develop different properties, thus in the gradual model the number of nodes was fixed in 2 and after a series of trials, 10 sublayers were set to constitute each one of the three regions as it can be seen in Fig. 1. Each sublayer is an EMA composed of c-Si, SiO_2_ and air with the porosity changing slightly from one sublayer to the next. With this approach, the inhomogeneous character of pSi samples of medium porosity (proportional ratio between the solid phase and air) has been well represented^7^. A system of air/gradual pSi/c-Si is formed in this way.

**Figure 2:**
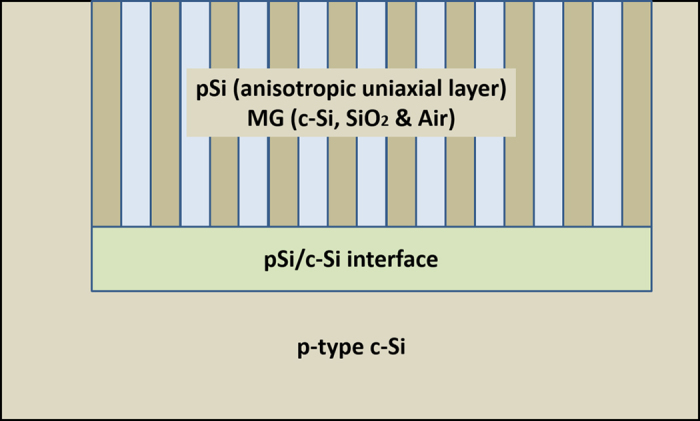
Scheme of the general structure of the morphological anisotropy model.

On the other hand, the anisotropic model introduces the column-like structure of pSi in the form of morphological uniaxial anisotropy because the pores running perpendicular to the surface of c-Si can generate directional dependence of the complex refractive index^9^. This model works better for samples of high porosity, where aligned pores of uniform size are present. In these kinds of samples, the effect of the pore growth mechanism produces an interfacial layer between pSi and c-Si, with different porosity (as will be seen in the micrographs shown later). The model constructed in this way is an air/uniaxial pSi/interface/c-Si system and is shown in Fig. 2. In this system, the mixture of c-Si, SiO_2_ and air is represented by the MG theory due to the low proportion of the solid phase (c-Si walls + SiO_2_ surface passivation) with respect to the air.

The effective medium approach used in each model was selected according to the porosity of the samples, i.e. for high porosity pSi the MG theory was applied and for low (or medium) porosity the Bruggeman theory was used. In both models, the fitting process was performed leaving the thickness and volume fraction (composition) of each layer free to be adjusted. The optical properties of each of the constituent materials (c-Si, SiO_2_ and air) are well known and were taken from the literature^16^.

## Results and Discussion

**Figure 3:**
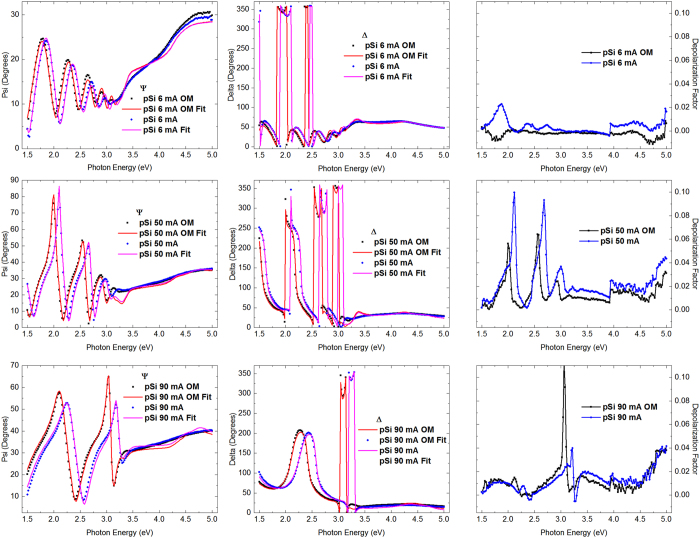
Ellipsometric ψ & Δ spectra of all the samples with and without OM in the electrochemical etching (solid symbols). The best fits are shown as continuous lines. The depolarization factor: deviation from the regular value of a not depolarizing sample = 1.0.

Figure 3a shows ellipsometric spectra of all the samples prepared with and without the addition of the OM to the electrolyte in their preparation. It can be seen that the SE measurements are sensitive to differences in the microstructure caused by variations in the electrochemical etching conditions. In the Figure, the depolarization factor is shown to keep in mind that light dispersion effects of a random pSi structure can lead to loss of accuracy of the ellipsometric measurements, in spite of the small size of the light spot used to measure (1 mm), which minimizes the depolarization sources^17^. As it can be observed, the higher the electrical current the stronger the depolarization effects and pSi prepared with 50.0 mA shows a mayor energy range of depolarized signal, as an evidence of a great inhomogeneity in the structure of these samples.

**Figure 4:**
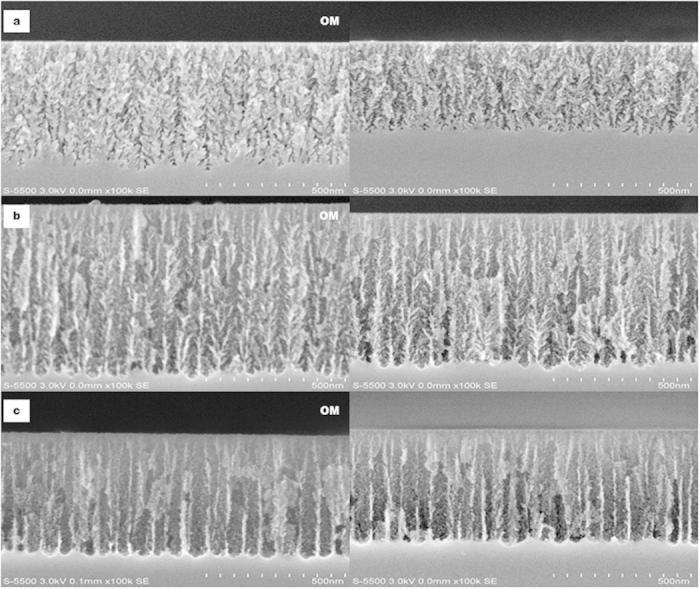
SEM images of pairs of samples obtained with secondary electrons at 100 k X. (**a**) 6.0 mA with and without OM, (**b**) 50.0 mA with and without OM, and (**c**) 90.0 mA with and without OM.

As it was mentioned in the previous section, the SEM micrographs were used to select the optical models to be applied in the data analysis of each sample. Figure 4 is a collection of micrographs showing the typical microstructures of pSi produced by using the electrolyte with and without the OM. In spite that the thickness of all the samples is comparable (500 nm, approx.), the differences in the porosity and the structure of the skeleton, caused by the change in the electrical current applied in the electrochemical process, can be seen. In the micrographs corresponding to 6.0 mA (Fig. 4a), the skeleton looks thick (the scale bar is 500 nm) and with dendritic-like structure. The structure of the 50 mA samples (Fig. 4b) is pretty much the same as the former case, but bigger pores were developed. While, the 90.0 mA electrical current (Fig. 4c) produces a very thin skeleton and pores running perpendicular to the surface of the substrate. When samples prepared with and without OM in the electrolyte at the same applied electrical current are compared, it cannot be notice any significant difference between them at this scale, in contrast to the deviations of the ellipsometric angles observed in Fig. 3.

The optical models constructed for the fitting process of each pair of pSi samples allowed to determine structural differences through the analysis of the fitted model parameters and the optical properties calculated from them. Table 1 shows the summary of the fitted parameters indicating the model approach, the EMT used and the pore walls material selected to represent each sample. The porosity and the effective complex refractive index were determined as a weighted average of each layer with respect to their corresponding thickness. Each gradual layer was taken as the average of their 10 constituents sublayers. In the case of the anisotropic layers, the ordinary and extraordinary contributions were averaged too. As it can be seen, for samples prepared with the highest electrical current (90.0 mA), the anisotropic model with MG and SiO_2_ as surface passivation gives the best fits (lowest values of the figure of merit based on the Levenberg-Marquardt algorithm, χ^2^). However, χ^2^ for the samples prepared with 50.0 mA are too high, likely because of the percolation limit of the MG approach is between 15 to 25% of the solid phase and the porosity of these samples is between 60 and 70%. Nevertheless, the comparative study between the samples prepared with and without the OM was carried out with the same model. On the other hand, for the samples prepared with the electrical current of 6.0 mA the optical model used was based on the gradual porosity approach with a three component EMA: c-Si, SiO_2_ and air. In this case, the best fits resulted in a zero volume fraction of the surface oxide. Besides the fact that it is difficult to differentiate between the optical properties of air and SiO_2_ in the range of photon energy studied here, the issue of the absence of surface oxide could be explained because of the lack of room for the oxide to growth in samples with low porosity (pore sizes of few nanometers).

**Figure 5:**
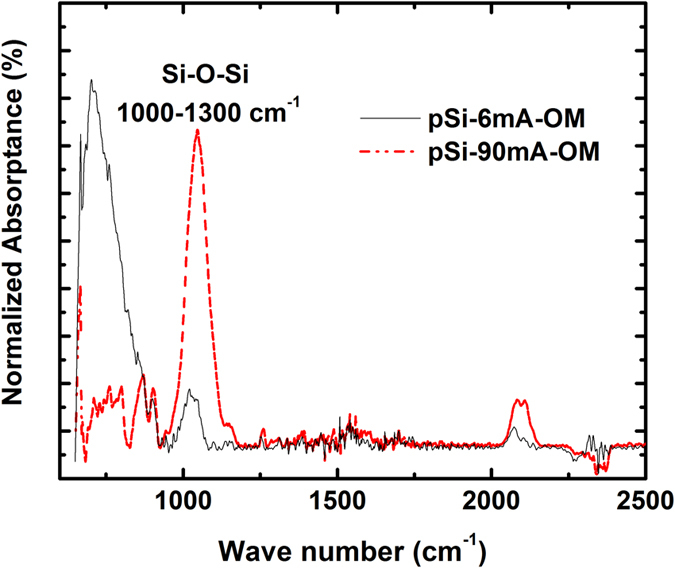
IR spectra of samples prepared with 6.0 and 90.0 mA. Both samples obtained with OM.

The infrared spectroscopy spectra of the samples produced at 6.0 and 90.0 mA is shown in Fig. 5. As it can be observed, the SiO_2_ content of samples produced with 6.0 mA is not zero, but it is significantly lower than the corresponding to the sample produced with 90.0 mA. This is true in both cases, with and without OM, but only one comparison is shown for simplicity.

**Figure 6:**
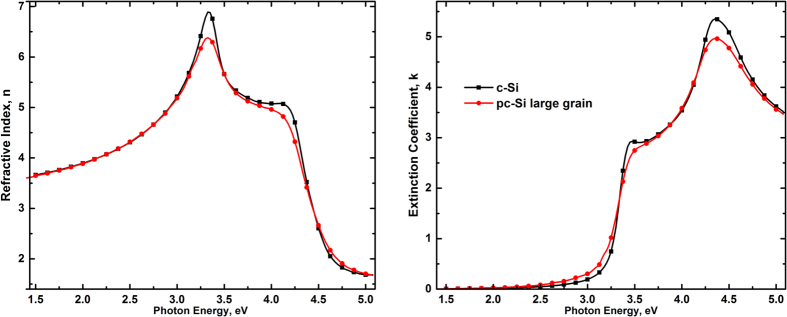
Optical properties (*n*
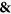
k) of c-Si and pclg-Si showing the effects caused by the decrease of the long range order in pclg-Si^18^.

Looking at the fitted parameters of each pair of samples it is possible to see some of the followed trends. For instance, in the case of the samples prepared with 90.0 mA, the porosity and the SiO_2_ content suggest that the presence of the OM in the electrolyte, induces oxide growth and thinning of the c-Si walls, in agreement with previous observations of the effect of the OM on pSi structures^5^. Here, we propose that the structure of the columns has changed. This interpretation is based on the comparison between fittings carried out for the same sample, changing the material used to represent the optical response of the walls, from c-Si in the initial model to polycrystalline large grain silicon (pclg-Si) in the final model^14^, keeping unchanged all the others components. The sample with OM showed better fit when the walls were represented with pclg-Si than with the model with c-Si. On the other hand, the sample without OM showed the best fit when c-Si was used in the model. The optical properties of pclg-Si have proved to be very useful in describing the effects of the size reduction of the c-Si pore walls in pSi, because the decrease in long range order of both systems (the nanocrystalls represented by pclg-Si and the nanometric c-Si pore walls) has similar effects on the optical response of silicon. Such effects can be observed in Fig. 6 where the optical spectra of c-Si and pclg-Si are shown. The broadening and the attenuation of the features (representing interband electronic transitions) of the pclg-Si optical response (~3.4 and 4.2 eV), compared with those of c-Si, are due to the decrease in the long range order^14^. In addition, these results suggest that the effect of the size reduction of the c-Si walls on the optical response of pSi is more significant than the degree of surface oxidation, opposite to what was proposed by Lugo *et al*.^13^.

With respect to the lower electrical currents section of Table 1 (50.0 and 6.0 mA), the results show that the effect of the OM is less prominent than in the case of the samples prepared with the highest current. As it can be noticed in the table, the values of porosity are very close to each other for samples with and without the OM at both electrical currents, 50.0 and 6.0 mA. Moreover, the SiO_2_ content results very similar too (zero in the case of 6.0 mA). These observations suggest the existence of a threshold value of porosity at which the OM can effectively modify the pSi skeleton, under the conditions established in this work for the fabrication of the samples.

To support this observation, we performed the spectro-ellipsometric analysis to two additional pairs of samples prepared with 60.0 and 75.0 mA. The summary of the results is shown in Table 2. As can be seen, the thickness, the porosity and the volume fraction of the samples are very close among the samples with and without OM at each current applied. These results support the observation of a porosity threshold for the action of the OM over the structure of pSi, under the conditions studied here.

**Figure 7:**
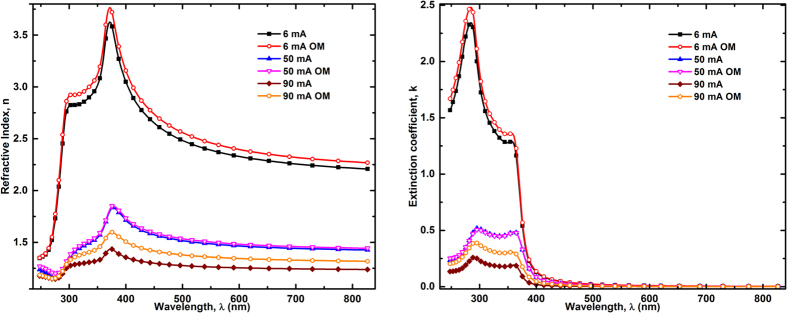
Refractive index spectra of all pSi samples as a function the applied electrical current.

Figure 7 shows *n*&*k* spectra obtained from the fitted parameters of all the pSi structures studied here. As it can be seen, the optical properties of all the samples are well behaved and comparable with the typical values reported in the literature^4^. In addition, the *n*&*k* spectra of samples prepared with and without the OM in the electrolyte at electrical currents of 50.0 and 6.0 mA did not show significant differences. Comparatively, in pSi prepared with 90.0 mA, the effect of the difference in porosity and oxide content between the samples with and without the OM results evident.

The *n* values for the samples prepared at the lowest electrical current in the region of transparency (above 500 nm) indicate higher density of these layers, in agreement with their porosity. All the features of the c-Si optical response (see Fig. 6) appear attenuated in the *n*&*k* spectra of all the pSi samples, as an effect of the mixture with air and, in some cases, with SiO_2_ too. The broadening of the peaks at ~3.4 eV (364 nm) and 4.2 eV (295 nm) reveals wall size reduction effects in samples prepared with the highest current, as a direct result of using pclg-Si in the models.

**Figure 8:**
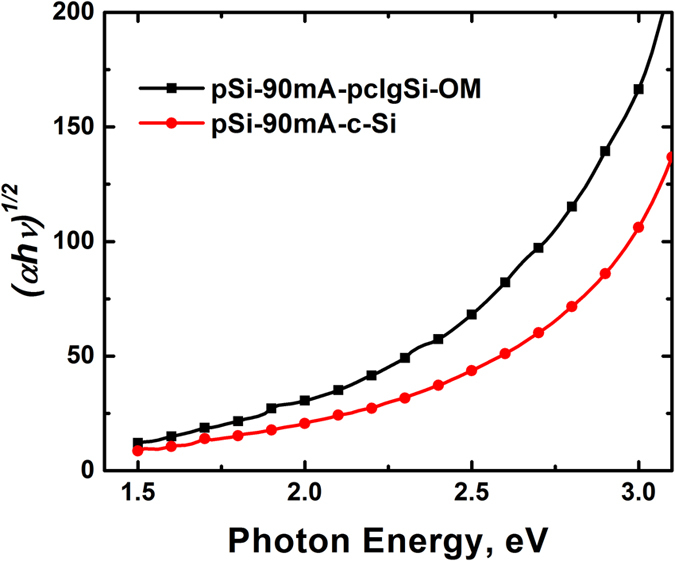
Indirect bandgap absorption coefficient of pSi samples prepared with 90.0 mA.

The absorption coefficient was determined from the *k* spectra of samples prepared at 90.0 mA, and it is shown in Fig. 8 in the form of *hν vs (αhν)*^*1/2*^ to identify possible changes in the bandgap of pSi due to the addition of the OM to the electrolyte. As it can be seen, the behaviour of the plots shows an increase of the absorption of the film prepared with OM in the electrolyte that is generally associated to an increase in the skeleton size, probably due to a lower porosity. We also clearly observe the plots for both samples departing from the lineal behavior usually observed in pSi produced from P^+^ silicon substrates (resistivity in the range of 10–2 Ω-cm). However, the curves cannot be directly associated to a transition due to a direct band gap but they are interpreted either in terms of a continuous band gap distribution or in terms of a large energy gap corresponding to the maximum slope, the low energy absorption tail being associated with a large defect concentration in the gap^19^.

## Conclusions

Current research on porous silicon includes the construction of complex structures with luminescent and/or photonic properties. Today, the preparation of pSi with both characteristics is still challenging and there are open questions related with the different pSi optical properties according to fabrication conditions. In spite of the difficulty to construct a general model to describe the optical response of pSi, due to the great variety of microstructures produced by different electrochemical etching conditions, most of the cases studied here fall into the scope of the two proposed models and these models yield parameters to calculate optical and structural properties. The methodology adopted in this work to determine structural and optical properties of pSi results in a very close approximation of its optical response. Throughout the analysis of the pSi ellipsometric data we obtained good approximations of the UV-Vis-NIR optical properties (*n*&*k*) of samples prepared with and without OM and correlated such properties with the particular microstructure of each sample. These results help us to understand the effect of the OM in the production process, which modifies the thickness and surface passivation of the pores and can be used as design parameters for the development of complex structures based on pSi.

## Additional Information

**How to cite this article**: Montiel-González, Z. *et al*. Role of an Oxidant Mixture as Surface Modifier of Porous Silicon Microstructures Evaluated by Spectroscopic Ellipsometry. *Sci. Rep*. **6**, 24798; doi: 10.1038/srep24798 (2016).

## Figures and Tables

**Table 1:** Summary of the fitted model parameters.

**Sample, Anisotropic-MG, I (mA) ± 0.1%**	**J (mA/cm**^**2**^)	χ^**2**^	**SEM Thickness (nm)**	**SE Thickness (nm)**	**Porosity %**	**SiO**_**2**_ **Content %**
90 mA c-Si	59.21 ± 1.48	8.4	544.2	591.0 ± 5.8	78.8 ± 0.4	7.8 ± 0.75
90 mA OM pclg-Si	59.21 ± 1.48	6.3	597.4	583.8 ± 6.3	72.2 ± 0.4	12.0 ± 0.64
50 mA c-Si	32.89 ± 0.82	36.2	733.5	731.7 ± 15.3	64.9 ± 0.9	15.0 ± 1.75
50 mA OM c-Si	32.89 ± 0.82	36.5	711.7	755.6 ± 21.3	64.8 ± 0.8	14.8 ± 1.76
**Sample, Gradual-EMA**		χ^**2**^	**SEM Thickness (nm)**		**SE Thickness (nm)**	**Porosity %**
6 mA c-Si	3.94 ± 0.09	4.9	442.1		420.9 ± 5.2	50.6 ± 0.6
6 mA OM c-Si	3.94 ± 0.09	9.8	458.1		425.2 ± 8.8	48.7 ± 0.9

**Table 2:** Summary of the fitted model parameters for the supporting samples prepared with 60.0 and 75.0 mA.

**Sample, Anisotropic-MG, I (mA)** ± **0.1%**	**J (mA/cm**^**2**^)	χ^**2**^	**SEM Thickness (nm)**	**SE Thickness (nm)**	**Porosity %**	**SiO**_**2**_ **Content %**
60 mA pclg-Si	39.47 ± 0.98	18.9	450.0	472.76 ± 7.49	57.55 ± 0.78	14.71 ± 0.84
60 mA OM pclg-Si	39.47 ± 0.98	29.76	440.0	471.25 ± 3.94	57.11 ± 0.34	13.42 ± 0.09
75 mA pclg-Si	49.24 ± 1.23	13.57	450.0	477.90 ± 6.73	61.13 ± 0.60	14.0 ± 0.64
75 mA OM pclg-Si	49.24 ± 1.23	14.85	450.0	470.62 ± 6.32	60.77 ± 0.61	13.57 ± 0.63
